# Round gobies (*Neogobius melanostomus*) in the River Rhine: Population genetic support for invasion via two different routes

**DOI:** 10.1371/journal.pone.0310692

**Published:** 2024-09-19

**Authors:** Kathrin P. Lampert, Lisa Heermann, Svenja Storm, Philipp Emanuel Hirsch, Alexander F. Cerwenka, Katja Heubel, Jost Borcherding, Ann-Marie Waldvogel

**Affiliations:** 1 Institute of Zoology, University of Cologne, Köln, Germany; 2 Ecological Field Station Rees, Institute of Zoology of the University of Cologne, Rees, Germany; 3 Landesfischereiverband Westfalen und Lippe e.V., Münster, Germany; 4 Department of Environmental Sciences, Program Man-Society-Environment, University of Basel, Basel, Switzerland; 5 SNSB–Zoologische Staatssammlung München Münchhausenstraße 21, München, Germany; 6 Research and Technology Centre West Coast (FTZ), Kiel University, Büsum, Germany; University of Basrah, IRAQ

## Abstract

The round goby, *Neogobius melanostomus*, is a successful invasive fish species. Originating from the Caspian and Black Sea, it is now distributed widely within European fresh- and brackish waters. The River Rhine was colonized in 2008 only a few years after the opening of the Rhine-Main-Danube canal and only four years after *N*. *melanostomus* was first reported in the upper Danube River. Its invasion history of the River Rhine was unclear because the species was first detected close to the Rhine river delta which would suggest a route of invasion other than via the Rhine-Main-Danube canal. To investigate the colonization history of *N*. *melanostomus* in the Rhine, we combined abundance estimates with molecular analysis. Abundance estimates found *N*. *melanostomus* to be dominant in the Lower Rhine (> 90% of all catches). Molecular analysis was done on 286 individuals from four different sites. Using the mitochondrial control region (d-loop), we found three different haplotypes in both Rhine sites. None of the potential invasive source populations in the rivers Danube and Trave exhibited all three haplotypes. The molecular data therefore supported a scenario of two different colonization directions. Our results show that the invasion history of the River Rhine is complex and warrants further investigation.

## Introduction

Climate change, habitat loss and fragmentation, and the introduction and spread of invasive species constitute the three main threats to the planet’s environmental stability [1]. Invasive species can have detrimental effects on biodiversity [2] and the genetic diversity of native species [3] and can alter food webs as well as the physical and abiotic properties of invaded ecosystems [4,5]. Invasive species’ contribution to global animal extinctions is estimated to be 33% [6]. Freshwater ecosystems, naturally embedded in land masses and therefore prone to human and socioeconomic impacts, have an elevated risk of negative ecological impacts by invasive species [7].

The round goby, *Neogobius melanostomus* (Perciformes: Gobiidae; Pallas, 1814), is a widely distributed invasive species in temperate fresh- and brackish waters of the northern hemisphere [8]. It is a benthic fish native to the brackish waters of the Caspian and Black Seas of the Eurasian continent and has spread throughout the North American Great Lakes and Europe (overview in [9]). Invaded ecosystems may suffer a population decline of native fish species as well as a decrease in the diversity of invertebrate organisms [10,11].

*N*. *melanostomus* has several traits that allow for successful invasion. As a possible competitor with native fishes, *N*. *melanostomus* has a broad diet and is often aggressive towards other fish species [12,13]. It has high reproductive success, expands it range rapidly and often alters trophic dynamics due to predation and competition [12,14–17]. *N*. *melanostomus* individuals can live up to four years with males first reproducing at an age of one to two years, females reach reproductive age after at least two years. Round gobies have a high fecundity and can spawn multiple times during their long breeding season from April to September [13,18]. Reproductive output is enhanced by parental care provided by the males that guard and fan the eggs [19].

*N*. *melanostomus* in all live stages can be transported in ballast water, which is used to provide stability and maneuverability to ships, and which is later disposed into channels and ports far from its origin [20,21]. Especially early juveniles are being picked up by ballasting ships because they are nocturnal and feed on pelagic zooplankton close to the water surface where they can be present in large densities [22]. In addition, the eggs are sticky and could potentially be adhered to the hull of ships, increasing invasion incidents [23].

*N*. *melanostomus* has a broad spectrum of tolerance to changing and/or adverse environments, with the ability to survive and move in man-made navigation channels, where fluctuations in depth, temperature and salinity are extremely pronounced [8]. Experiments have shown that *N*. *melanostomus* can be transported and survive directly in salinities from 0 to 20 ppt without acclimation, which points to its high adaptability [24].

In the Baltic Sea, *N*. *melanostomus* was first recorded in 1990 and has since then spread considerably [25]. European rivers were colonized around 2000 [8]. In the Danube River, *N*. *melanostomus* moved upstream from its original downstream and estuary range and reached the Upper Danube in 2004, where it became the most abundant fish species just 10 years after its first introduction [15]. The Rhine invasion started shortly afterwards. In the Lower Rhine *N*. *melanostomus* was reported in 2008 [10] and in the Upper Rhine it was reported in 2011 [26]. Today colonization extends throughout most of the river. With a length of 1,320 km and a basin of 185,000 km^2^, the River Rhine is one of the largest freshwater ecosystems in Western Europe. It empties into the North Sea and connects to the Black Sea through the Rhine-Main-Danube canal, thus being an important waterway within the European Union. Historically, the Rhine suffered severe wastewater pollution and even catastrophic chemical pollution events (review in [27]). Due to large conservation efforts the water quality improved enormously since the 1980s, however, the ecosystem’s community equilibrium is still delicate and prone to invasions [28].

**Fig 1:**
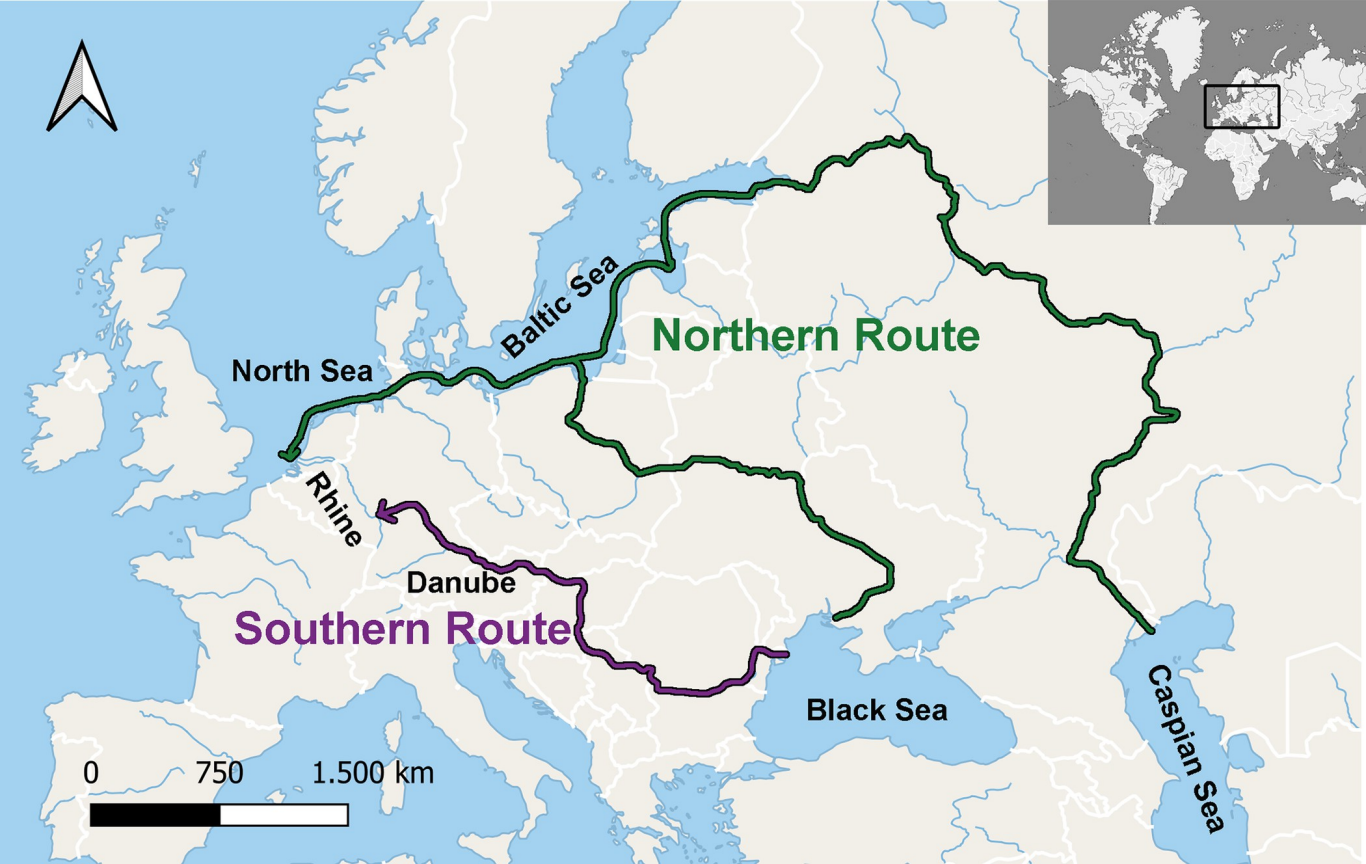
Potential colonization routes of *Neogobius melanostomus* [10,32].

Intensive ship traffic in combination with the high invasion potential of *N*. *melanostomus*, make a single invasion event unlikely. In the Dutch Rhine delta, the species was first recorded in 2004 [29], likely being introduced via shipping from the Baltic Sea [30]. Almost concurrently, *N*. *melanostomus* was recorded in the upper Danube [31] and there the spread to the Rhine via the Rhine-Main-Danube channel was predictable. Borcherding et al. (2011) [10] suggested the following two potential invasion routes (Fig 1) as equally probable, when anglers first discovered *N*. *melanostomus* in the Lower Rhine in 2008:

1. Invasion from the Caspian Sea to the Baltic Sea, through the channels of the Volga River and the Volga-Baltic channel, from where the goby reached the North Sea, subsequently entering the Rhine and colonizing it upstream. (Northern Route)
2. Invasion from the Caspian or Black Sea upstream the Danube River until reaching the Rhine River through the Main-Danube Channel, following the course downstream of the Rhine. (Southern Route)

Established populations of invasive species are characterized by the ecological, phenotypic and genetic properties of their origin-population plus any phenotypic and genetic changes resulting from or following the invasion event itself [33] (recent examples e.g. [34,35]). Populations that belong to the same invasive species but have arrived via different introduction routes may differ genetically and morphologically and/or ecologically.

The objective of this study was to investigate round goby invasion in the Rhine River using genetic markers and temporal abundance estimates. Specifically, we compared genetic markers of *N*. *melanostomus* in the area where *N*. *melanostomus* was first reported (Lower Rhine Rees) with those from potential source populations representing the different invasion routes: Trave (Baltic Sea–Northern invasion route) and Danube (Southern invasion route). While mitochondrial markers had, so far, revealed low levels of differentiation in European *N*. *melanostomus* and were insufficient to distinguish between source populations [36,37], nuclear markers had revealed much higher levels of diversity promoting multiple invasions events [38]. We therefore combined the advantages of mitochondrial DNA inheritance (conservation of haplotypes due to lack of recombination) and a non-translated gene region (high variability) by investigating the mitochondrial dloop. If *N*.*melanostomus* invaded the River Rhine simultaneously via the two different routes, we predicted that the Rhine River round goby genetic markers would reflect genetic properties of both potential origin-populations (Trave and Danube rivers).

## Material & methods

### Ethics statement

All field collections were carried out following the local guidelines and regulations of the states and nations for handling invasive vertebrate species. All gobiids were euthanized using MS-222. Lower Rhine angling permits were provided by the Rheinfischereigenossenschaft to Jost Borcherding. Permissions for angling fish in the Upper Rhine were obtained from Swiss cantonal authorities and fishing rights owners of the Swiss cantons Basel-Stadt and Aargau and were filed under the numbers: GS-18-07-01, 2017/14, 2-3-6-4-1. Gobies from the Trave were obtained in conjunction with specimen supply for experiments at Hamburg university–angling permit nr 59/16 from Amt für Vebraucherschutz, Veterinärwesen und Lebensmittelüberwachung, Hamburg. In the Donau fish were caught under the permission of the local fisheries administration (Fischereifachberatung Niederbayern) via electrofishing. Electrofishing was conducted under license number 31-7563/2 to the Aquatic Systems Biology Unit, Technische Universität München. All required qualifications of the involved people (fishing licenses, electrofishing certificates, animal welfare training) were valid and formally approved.

### Field sampling

*N*. *melanostomus* of the Lower Rhine were captured once a year (July) by angling at a groin field close to the city of Rees (Rhine km 842, cf. [10]). Sitting at the edge of the groin, anglers were assigned to sample at either of the two local habitat types: rip-rap or sand. Two anglers directly exposed their bait upon the rip-rap structure only 1 m from the water line (fishing with a float); in the following this station is named “rip-rap”. Two other anglers exposed their bait roughly 15–20 m in front of the groin in deeper waters on sandy bottom close to the main current of the main stream (fishing with bottom lead); in the following this station is always named “sand”. All anglers used hooks of the size 10–14 and baited regularly with 1–3 maggots.

Each catch was directly noted with angler name, original habitat (rip-rap or sand), exact time code, species and total length (TL, measured to the nearest 1 mm). In addition, all gobies were sexed, sampled in buckets (maximum for one hour, separated in rip-rap and sand), then anesthetized using MS222 (Sandoz) and stored on ice and shortly later conserved at a temperature of -18°C. Sampling took place from 2010 until 2020, and each angling survey lasted on average 8 hours mainly during daytime. At any time during each survey, the name of the anglers and their exact fishing time was noted as the basis to calculate quantitative data as catch per unit effort (CPUE). All catches from each year were summed up and calculated as CPUE (catch per rod per hour).

To compare *N*. *melanostomus* from the Lower Rhine with *N*. *melanostomus* from potential source populations, we got additional specimens from the upper Rhine (Basel), the upper Danube River (Deggendorf and Passau) as a representative for the Southern Route genotypes and from the Trave River close to the Baltic Sea representing the Northern Route genotypes. Fish in the upper Rhine and the Trave were also collected by angling, fish from the Danube were caught by electrofishing (S1 Table).

### Molecular analyses

Fin clips stored in ethanol were used for genotyping. DNA was extracted using the Qiagen DNeasy kit according to the manufacturer’s recommendations. Because earlier analyses with the COI and *CYTB* mitochondrial genetic markers found little haplotype diversity in Western European round gobies [36,37], we used the more variable d-loop (= control) region of the mitochondrial genome. To amplify the d-loop region we used the primers fwd2 and rev2 from Adrian-Kalchhauser et al. (2017) [39]. The PCR reaction was performed in a final volume of 12.5μL containing 6.25μL 2xRedTaq Master Mix 1.5mM MgCl_2_ (VWR Life Science, Darmstadt, Germany), 1μL of each primer (10μM, Metabion, Planegg, Germany) and 4.25μL of template DNA. PCR conditions were: 94°C 3 min initial denaturation, 40 cycles of 94°C 30s, 56°C 30s, 65°C 2min, followed by a final elongation step at 65°C of 5min. PCR products were Sanger sequenced in forward and reverse direction at Eurofins Genomics Europe (Ebersberg, Germany). Sanger sequences were evaluated, edited and a consensus sequence for each individual was produced using the program BioEdit [40]. All consensus sequences were aligned using the ClustalW algorithm implemented in BioEdit. The alignment was visually inspected and algorithm errors due to repetitive regions were corrected manually. Only sequence variants that appeared more than five times in the dataset were categorized as separate haplotypes.

The haplotype distributions (A versus non A = B+C) among sample groups were compared using a chi^2^ or Fisher’s exact test. Chi^2^ tests were used for comparisons of samples where less than 20% of expected values were below 5 (sites, sex, habitat) using the program (PAST vers.4.03). Fisher’s exact test was used if more than 20% of expected values were below 5 (year) using the astatsa website (https://astatsa.com/FisherTest/). To test if the proportion of B+C alleles increased or decreased with time a binomial generalized linear model (GLM) analysis was used (PAST vers. 4.03). Bonferroni correction was applied to correct for multiple testing.

## Results

**Fig 2:**
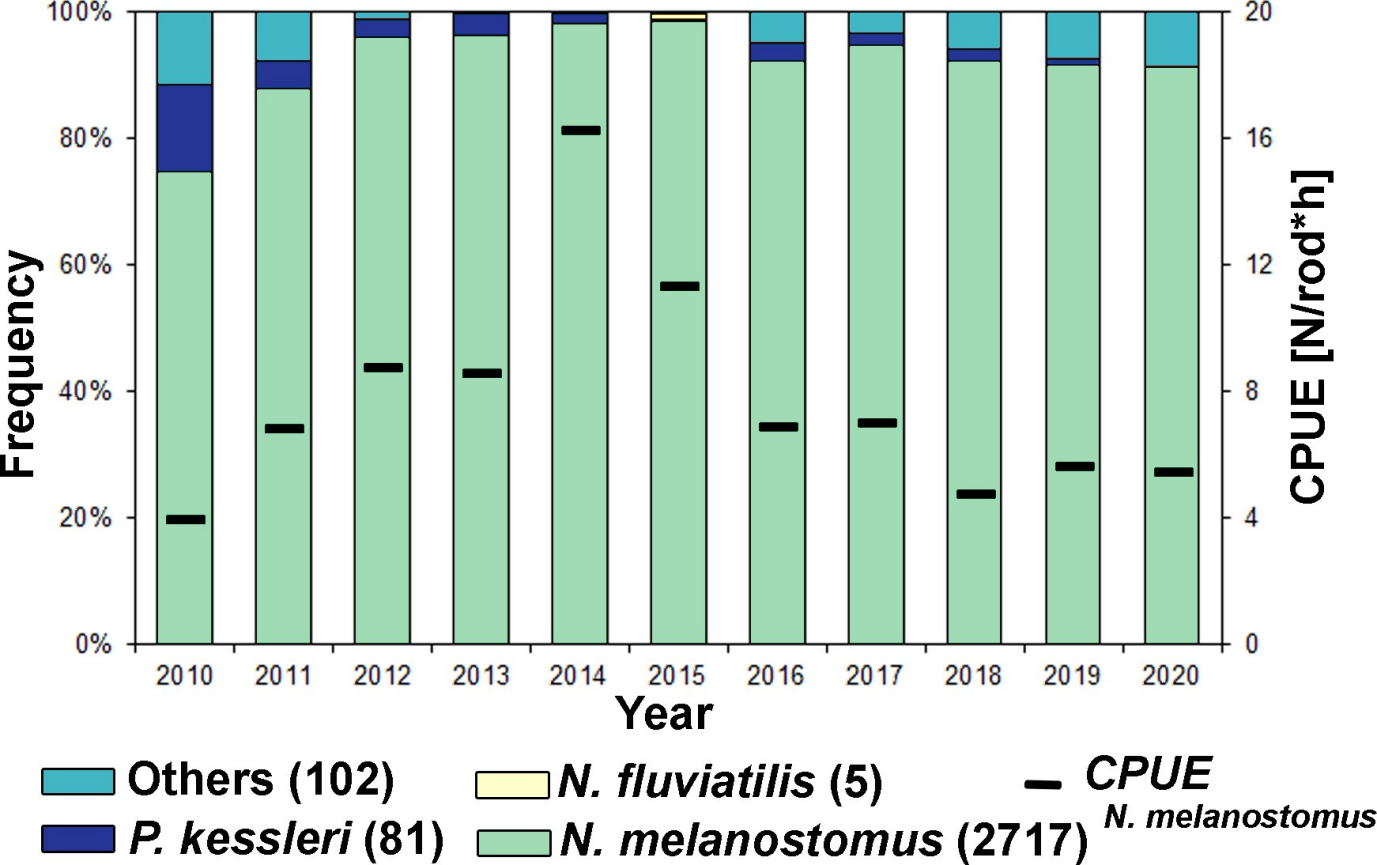
Frequency of different goby species (left-bound axis) and catch per rod per hour (CPUE) of *N*. *melanostomus* (right-bound axis) in the Lower Rhine (km 845, Rees) of annual angling events from 2010 to 2020. Numbers in brackets after species’ names gives the total number of individuals caught.

Fish sampling efforts in the Lower Rhine between 2010 and 2020 resulted in a collection of 2905 gobiids. The most abundant species was *Neogobius melanostomus* (93.5%) (Fig 2). *N*. *melanostomus* numbers were highest in 2014 when 16 fish per rod per hour were caught. Captured gobies had an average length of 86.9 mm +/- 17.06 mm standard deviation.

**Fig 3:**
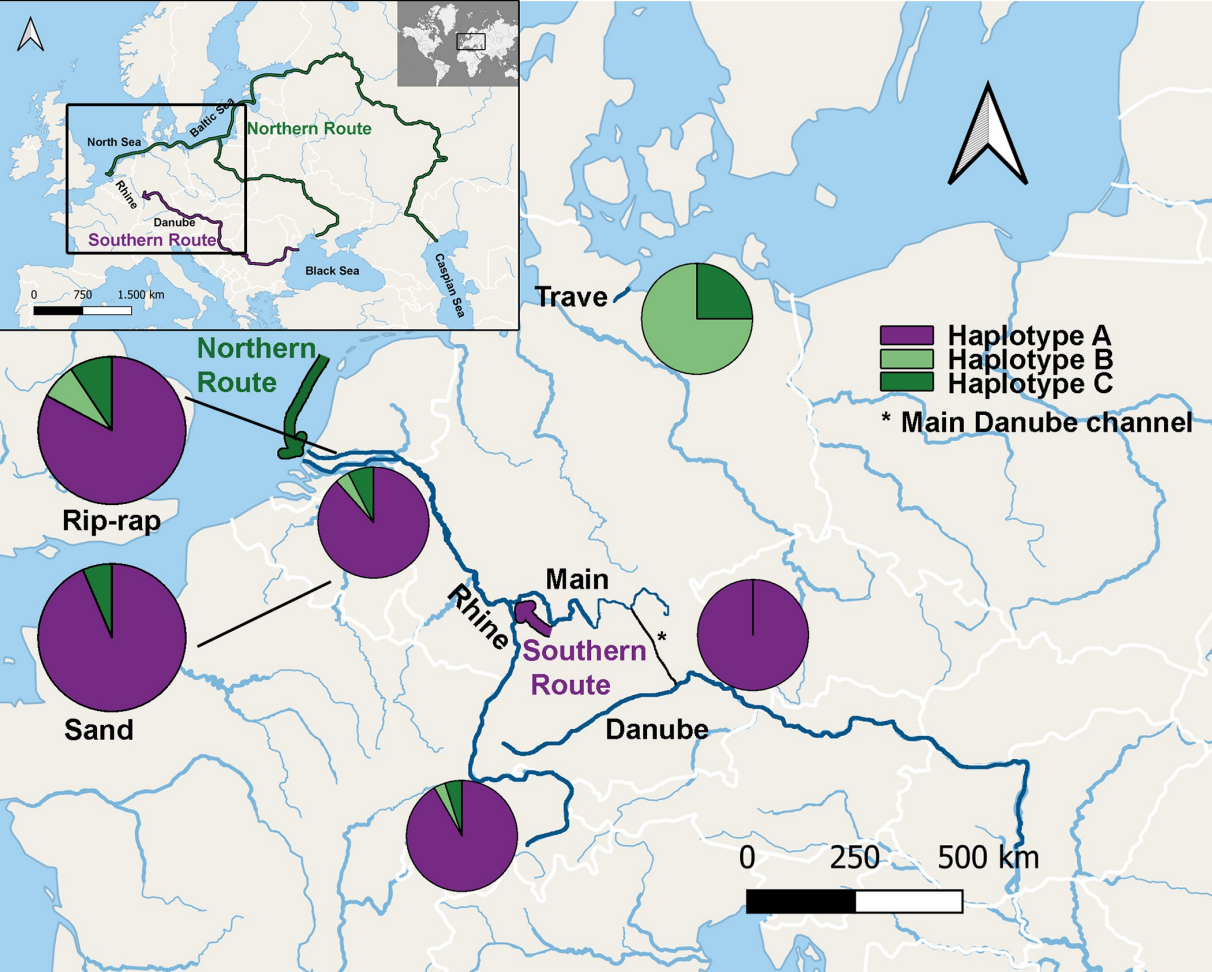
Summary of haplotype distribution in different sites. In the Lower Rhine site habitat specific haplotype distribution is also shown. Rivers relevant in the study are marked in darker blue, the Main Danube channel is shown in black (*).

A total number of 286 individuals were genotyped (146 from the Lower Rhine (Rees), 60 from the Upper Rhine (Basel), 60 from the Danube and 20 from the Trave (Fig 3, details in S1 Table). Three haplotypes were found (A, B and C) that differed in a number of bases and deletion/insertions sites (GenBank Accession no. PP342297-PP342299, Table 1, full length alignment S2 Table).

**Table 1:** Haplotype sequence differences. (H–haplotype).

	Position in alignment															
**H**	22	86	96–101	105	106	175/176	340	407	409	412	415	466	478	539	554	649
**A**	A	A	------	T	-	AT	G	C	T	A	T	A	C	C	-	A
**B**	A	A	------	T	T	--	A	T	C	G	C	G	T	C	-	T
**C**	C	T	ATATAT	C	T	--	A	T	C	G	C	A	T	T	T	T

Haplotype frequencies varied substantially between sites (chi^2^ = 134.67 df = 3 p < 0.00001 (Bonferroni corrected p = 0.0125)). While the samples from the Danube only exhibited haplotype A, individuals from the Trave exclusively had haplotypes B and C. The samples from the Rhine (Rees and Basel), however, carried all three haplotypes with A being the most common haplotype and B and C at lower frequencies.

**Fig 4:**
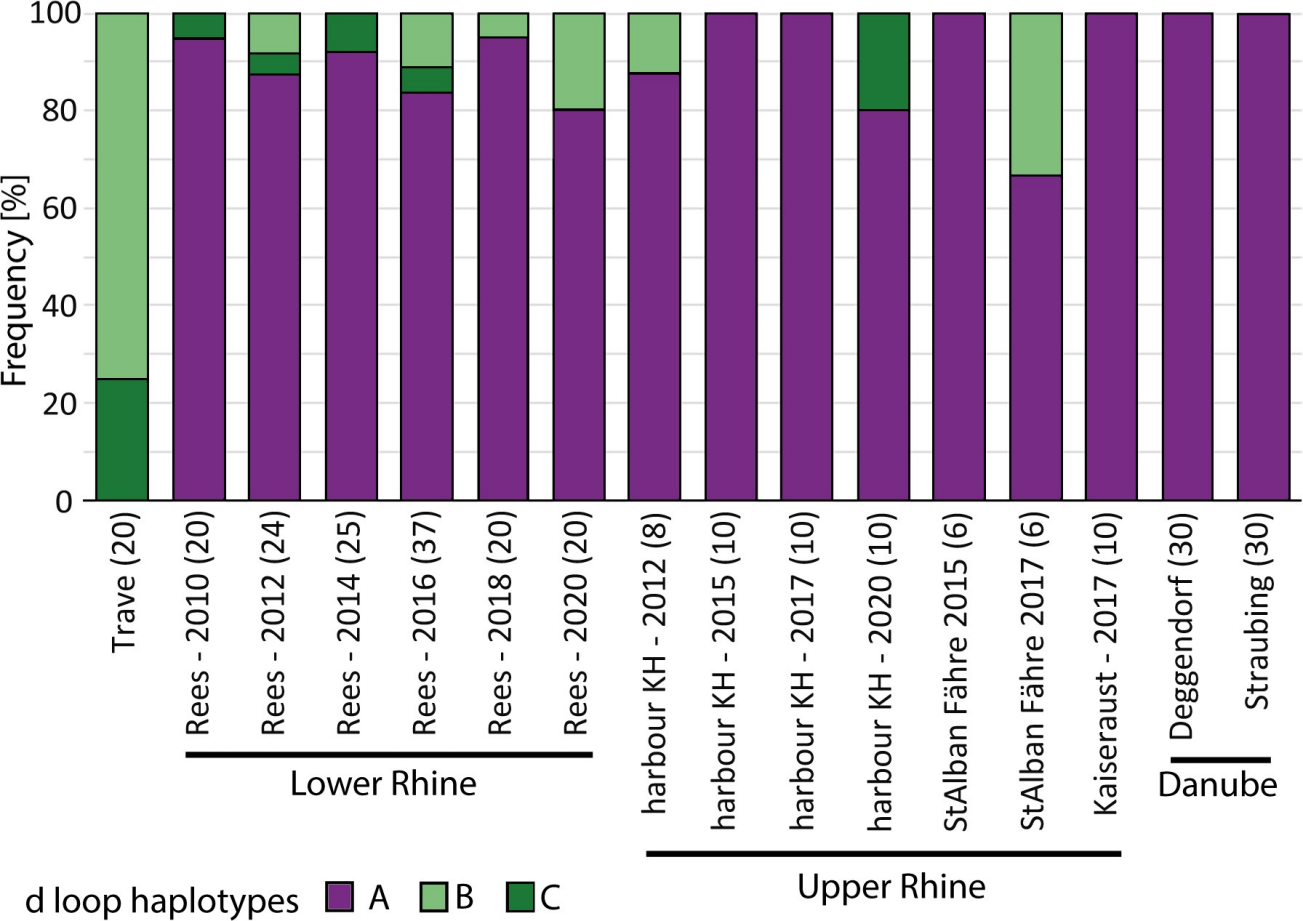
Detailed haplotype distribution in different sites and years (numbers of analyzed individuals are given in brackets). (Upper Rhine sites are situated within a 15km river stretch within Basel. River distance between Deggendorf and Straubing is approximately 24km).

Haplotypes of gobies caught in the Lower Rhine population did not show a habitat (rip-rap or sand) specific distribution pattern (chi^2^ = 0.357 df = 1 p = 0.55, Fig 3, Table 2) and no significant difference in haplotype distribution (A versus B + C) could be detected between the sexes (chi^2^ = 0.0519 df = 1 p = 0.82) (Table 2). Haplotype occurrence stochastically varied between years; however, variation was random and did not show a specific pattern e.g. increase or decrease with time (Fisher’s exact test p = 0.282), GLM: Slope a: 0,071549 Std. err. a: 0,40007 Intercept b: -146,36 Std. err. b: 806,41, Log likelihood: -0,1739, G: 0,032517 p(slope = 0): 0,8569, Fig 4).

**Table 2:** Rees’ haplotype distribution summary.

Rees—sex (all years)			
	A	B	C
**male**	94 (88.8%)	4 (3.7%)	8 (7.5%)
**female**	36 (90%)	2 (5%)	2 (5%)
**Rees–habitat (2012–2020 both sexes)**			
	**A**	**B**	**C**
**Rip-rap**	53 (83%)	5 (7.8%)	6 (9.2%)
**Sand**	58 (93.5%)	0	4 (6.5%)

## Discussion

The primary goal of our study was to test the hypothesis that *N*. *melanostomus* invaded the River Rhine via two different routes using molecular markers and abundance estimates. We found *N*. *melanostomus* to be the dominant goby species in the Rhine with a 2014 abundance peak. The molecular analysis of the dloop revealed a mixture of three different haplotypes (A, B, C) in both Rhine sites. None of the potential source populations included all three haplotypes, instead the Danube population exclusively showed a single haplotype (A) and the Trave population comprised only individuals with the other two haplotypes (B, C). The molecular data therefore clearly supported a Rhine invasion via two different routes.

Systematic goby sampling in the Lower Rhine started in 2010, just a few years after the first reports of *N*. *melanostomus* in the upper Danube River as well as in the Dutch Rhine delta [29,31]. Their abundance was high, just two years after its initial detection for the area: *N*. *melanostomus* constituted the numerical majority of the catches. This indicates the high propagule pressure (the quality, quantity, and frequency of invading organisms [41]) of *N*. *melanostomus* facilitating the successful spread in new areas and ecosystems [42,43].

The high abundance made several simultaneous invasions likely, however, the geographic origin of the large number of fishes was unclear. Unravelling the *N*. *melanostomus* invasion route in the River Rhine was particularly problematic, as round gobies were recorded in the river mouth and at the same time in the River Danube as part of the Rhine’s inland river network (via the Rhine-Main-Danube Channel, [29,31,44]), making both routes equally likely.

Molecular investigations of invasive *N*. *melanostomus* focused on invasion time, worldwide expansion, genotypic variability and on the species’ origin [36,45–48]. The standard mitochondrial genetic markers used in these studies, such as COI and *CYTB*, however, revealed only low levels of genotypic diversity in invasive round goby populations across Europe [37]. A single haplotype carried by all investigated individuals was discovered and the colonization history of the Rhine could not be resolved [37]. Analysis of nuclear markers found higher levels of genotypic diversity with Baltic populations being more diverse than the Danube [38]. Multiple invasions were proposed and the Danube was determined as most likely source population for the Rhine [38]. For our study we purposefully picked the mitochondrial d-loop region, thereby combining the advantages of both, mitochondrial DNA inheritance (conservation of haplotypes due to lack of recombination) and a non-translated gene region (high variability). As expected, we found the d-loop region of *N*. *melanostomus* to be more variable than the other mitochondrial genes. The haplotypes detected could clearly be assigned to their population of origin: (Danube only A, Trave only B + C). Our finding that the potential source populations belong to different non-overlapping molecular lineages, while a mix of these lineages was found in the invasive population at the Lower Rhine, supports the hypothesis that *N*. *melanostomus* has invaded the River Rhine via two different invasion routes [10].

Most round gobies in the Rhine belonged to haplotype A which matched those found in the Danube River. This indicates that either more individuals arrived via the Southern route (Rhine-downstream), or individuals from the Southern route were more successful in colonizing the Rhine as compared to individuals from the Northern route (Rhine-upstream). This finding is consistent with Janac et al. (2017) [37] and Green et al. 2021 [38] who also concluded that Danube round gobies played an important role in colonizing the Rhine. Northern haplotypes B and C were also observed in the Upper Rhine (Basel) and no significant difference in haplotype ratios were found compared to the Lower Rhine population (Rees). Interestingly, the Upper Rhine was colonized four years later than the Lower Rhine [26] pointing to different dispersal mechanisms. For both Rhine sites vessel transport is likely the main source of *N*. *melanostomus* invasion. Lower Rhine colonization, however, could have been facilitated by the downstream drift of juveniles [11,14]. In contrast, the patchy distribution of haplotype B and C in the Upper Rhine (Fig 4) may suggest mainly human mediated transport into the Upper Rhine area rather than active upstream migration of fish, which would have resulted in a more even haplotype distribution [11,49].

While we do have evidence that the River Rhine was invaded from two directions, our data do not resolve whether there was more than one invasion wave from each direction or how continuous or intense the invasion events have been. It seems however likely that active migration is an ongoing process. Even though ballast water, as a frequent mode of assisted introduction and dispersal, has to be treated against living organisms since 2017, connecting waterways are still open to vessels as well as fish.

While invasive populations may undergo founder events [50] which may reduce their genetic diversity in new areas, this might not be the case in successive invasions [33,51,52]. In fact, successive invasion may enable contact of formerly separated lineages therefore enabling admixture and enhancing genotypic diversity. In the case of invasive round gobies of the Lower Rhine, genetic variability may have resulted from different source populations and may have promoted invasion success. This might be true for traits facilitating long-distance migration and traits required in changing abiotic regimes. Known examples are oxygen consumption, osmoregulation and the immune system [24,53]. In *N*. *melanostomus* the innate immune gene region has been shown to be extended compared to other teleost species and might therefore provide a broader pathogen resistance [53]. The large mitochondrial genome [39] might also play a role in invasion success.

In conclusion, we could resolve the origin of the *N*. *melanostomus* populations in the Rhine and indeed prove invasion from two different directions. Many questions, however, remain and we are planning future studies into the development of *N*. *melanostomus* morphology as well as investigate the questions of invasion success and local adaptation by studying full genomes from multiple individuals in the near future.

## Supporting information

S1 Table Sample information. (DOCX)

S2 Table *Neogobius melanostomus*—dloop haplotypes alignment. (DOCX)
