# Supplementary material for: Round gobies (Neogobius melanostomus) in the River Rhine: Population genetic support for invasion via two different routes
Source: PLoS One. 2024 Sep 19;19(9):e0310692. doi: 10.1371/journal.pone.0310692 (PMC11412513; doi:10.1371/journal.pone.0310692)
Supplement: S1 Table — (DOCX) [file pone.0310692.s001.docx]

**Table S1: Sample information.** (na – information not available)

| **sample name** | **origin** | **GPS coordinates** | **sampling year** | **sex** | **size [mm]** | **haplotype** |
| --- | --- | --- | --- | --- | --- | --- |
| 10nm01m | Rees (lower Rhine) | 51°41'8.44"N 6°28'28.35"E | 2010 | male | na | A |
| 10nm02m | Rees (lower Rhine) | 51°41'8.44"N 6°28'28.35"E | 2010 | male | na | A |
| 10nm03m | Rees (lower Rhine) | 51°41'8.44"N 6°28'28.35"E | 2010 | male | na | A |
| 10nm04m | Rees (lower Rhine) | 51°41'8.44"N 6°28'28.35"E | 2010 | male | na | A |
| 10nm05m | Rees (lower Rhine) | 51°41'8.44"N 6°28'28.35"E | 2010 | male | na | A |
| 10nm06m | Rees (lower Rhine) | 51°41'8.44"N 6°28'28.35"E | 2010 | male | na | A |
| 10nm07f | Rees (lower Rhine) | 51°41'8.44"N 6°28'28.35"E | 2010 | female | na | A |
| 10nm08f | Rees (lower Rhine) | 51°41'8.44"N 6°28'28.35"E | 2010 | female | na | A |
| 10nm09f | Rees (lower Rhine) | 51°41'8.44"N 6°28'28.35"E | 2010 | female | na | A |
| 10nm10f | Rees (lower Rhine) | 51°41'8.44"N 6°28'28.35"E | 2010 | female | na | A |
| 10nm11m | Rees (lower Rhine) | 51°41'8.44"N 6°28'28.35"E | 2010 | male | na | A |
| 10nm12m | Rees (lower Rhine) | 51°41'8.44"N 6°28'28.35"E | 2010 | male | na | A |
| 10nm13m | Rees (lower Rhine) | 51°41'8.44"N 6°28'28.35"E | 2010 | male | na | A |
| 10nm14m | Rees (lower Rhine) | 51°41'8.44"N 6°28'28.35"E | 2010 | male | na | A |
| 10nm15f | Rees (lower Rhine) | 51°41'8.44"N 6°28'28.35"E | 2010 | female | na | A |
| 10nm16f | Rees (lower Rhine) | 51°41'8.44"N 6°28'28.35"E | 2010 | female | na | B |
| 10nm17f | Rees (lower Rhine) | 51°41'8.44"N 6°28'28.35"E | 2010 | female | na | A |
| 10nm18f | Rees (lower Rhine) | 51°41'8.44"N 6°28'28.35"E | 2010 | female | na | A |
| 10nm19f | Rees (lower Rhine) | 51°41'8.44"N 6°28'28.35"E | 2010 | female | na | A |
| 10nm20f | Rees (lower Rhine) | 51°41'8.44"N 6°28'28.35"E | 2010 | female | na | A |
| 12lf01 | Rees (lower Rhine) | 51°41'8.44"N 6°28'28.35"E | 2012 | female | 65 | C |
| 12lf02 | Rees (lower Rhine) | 51°41'8.44"N 6°28'28.35"E | 2012 | female | 64 | A |
| 12lf04 | Rees (lower Rhine) | 51°41'8.44"N 6°28'28.35"E | 2012 | female | 73 | A |
| 12lf08 | Rees (lower Rhine) | 51°41'8.44"N 6°28'28.35"E | 2012 | female | 100 | A |
| 12lf09 | Rees (lower Rhine) | 51°41'8.44"N 6°28'28.35"E | 2012 | female | 69 | A |
| 12lf10 | Rees (lower Rhine) | 51°41'8.44"N 6°28'28.35"E | 2012 | female | 80 | A |
| 12lm02 | Rees (lower Rhine) | 51°41'8.44"N 6°28'28.35"E | 2012 | male | 96 | A |
| 12lm05 | Rees (lower Rhine) | 51°41'8.44"N 6°28'28.35"E | 2012 | male | 80 | A |
| 12lm07 | Rees (lower Rhine) | 51°41'8.44"N 6°28'28.35"E | 2012 | male | 104 | A |
| 12lm10 | Rees (lower Rhine) | 51°41'8.44"N 6°28'28.35"E | 2012 | male | 82 | A |
| 12sf01 | Rees (lower Rhine) | 51°41'8.44"N 6°28'28.35"E | 2012 | female | 83 | A |
| 12sf03 | Rees (lower Rhine) | 51°41'8.44"N 6°28'28.35"E | 2012 | male | 60 | A |
| 12sf04 | Rees (lower Rhine) | 51°41'8.44"N 6°28'28.35"E | 2012 | female | 81 | B |
| 12sf05 | Rees (lower Rhine) | 51°41'8.44"N 6°28'28.35"E | 2012 | female | 71 | A |
| 12sf07 | Rees (lower Rhine) | 51°41'8.44"N 6°28'28.35"E | 2012 | female | 85 | A |
| 12sf09 | Rees (lower Rhine) | 51°41'8.44"N 6°28'28.35"E | 2012 | female | 78 | A |
| 12sf14 | Rees (lower Rhine) | 51°41'8.44"N 6°28'28.35"E | 2012 | female | 74 | A |
| 12sm01 | Rees (lower Rhine) | 51°41'8.44"N 6°28'28.35"E | 2012 | male | 85 | C |
| 12sm02 | Rees (lower Rhine) | 51°41'8.44"N 6°28'28.35"E | 2012 | male | 86 | A |
| 12sm03 | Rees (lower Rhine) | 51°41'8.44"N 6°28'28.35"E | 2012 | male | 100 | A |
| 12sm06 | Rees (lower Rhine) | 51°41'8.44"N 6°28'28.35"E | 2012 | male | 102 | A |
| 12sm15 | Rees (lower Rhine) | 51°41'8.44"N 6°28'28.35"E | 2012 | male | 96 | A |
| 12sm19 | Rees (lower Rhine) | 51°41'8.44"N 6°28'28.35"E | 2012 | male | 100 | A |
| 12sm22 | Rees (lower Rhine) | 51°41'8.44"N 6°28'28.35"E | 2012 | male | 90 | A |
| 14lm01 | Rees (lower Rhine) | 51°41'8.44"N 6°28'28.35"E | 2014 | male | 92 | A |
| 14lm02 | Rees (lower Rhine) | 51°41'8.44"N 6°28'28.35"E | 2014 | male | 100 | A |
| 14lm03 | Rees (lower Rhine) | 51°41'8.44"N 6°28'28.35"E | 2014 | male | 90 | A |
| 14lm04 | Rees (lower Rhine) | 51°41'8.44"N 6°28'28.35"E | 2014 | male | 80 | A |
| 14lm05 | Rees (lower Rhine) | 51°41'8.44"N 6°28'28.35"E | 2014 | male | 102 | A |
| 14lm11 | Rees (lower Rhine) | 51°41'8.44"N 6°28'28.35"E | 2014 | male | 113 | A |
| 14lm12 | Rees (lower Rhine) | 51°41'8.44"N 6°28'28.35"E | 2014 | male | 93 | A |
| 14lm13 | Rees (lower Rhine) | 51°41'8.44"N 6°28'28.35"E | 2014 | male | 114 | A |
| 14lm14 | Rees (lower Rhine) | 51°41'8.44"N 6°28'28.35"E | 2014 | male | 94 | A |
| 14lm15 | Rees (lower Rhine) | 51°41'8.44"N 6°28'28.35"E | 2014 | male | 75 | A |
| 14lm16 | Rees (lower Rhine) | 51°41'8.44"N 6°28'28.35"E | 2014 | male | 95 | A |
| 14lm17 | Rees (lower Rhine) | 51°41'8.44"N 6°28'28.35"E | 2014 | male | 72 | A |
| 14lm18 | Rees (lower Rhine) | 51°41'8.44"N 6°28'28.35"E | 2014 | male | 112 | A |
| 14sm01 | Rees (lower Rhine) | 51°41'8.44"N 6°28'28.35"E | 2014 | male | 70 | B |
| 14sm02 | Rees (lower Rhine) | 51°41'8.44"N 6°28'28.35"E | 2014 | male | 71 | B |
| 14sm04 | Rees (lower Rhine) | 51°41'8.44"N 6°28'28.35"E | 2014 | male | 85 | A |
| 14sm06 | Rees (lower Rhine) | 51°41'8.44"N 6°28'28.35"E | 2014 | male | 73 | A |
| 14sm12 | Rees (lower Rhine) | 51°41'8.44"N 6°28'28.35"E | 2014 | male | 89 | A |
| 14sm13 | Rees (lower Rhine) | 51°41'8.44"N 6°28'28.35"E | 2014 | male | 103 | A |
| 14sm14 | Rees (lower Rhine) | 51°41'8.44"N 6°28'28.35"E | 2014 | male | 82 | A |
| 14sm15 | Rees (lower Rhine) | 51°41'8.44"N 6°28'28.35"E | 2014 | male | 101 | A |
| 14sm16 | Rees (lower Rhine) | 51°41'8.44"N 6°28'28.35"E | 2014 | male | 105 | A |
| 14sm17 | Rees (lower Rhine) | 51°41'8.44"N 6°28'28.35"E | 2014 | male | 95 | A |
| 14sm18 | Rees (lower Rhine) | 51°41'8.44"N 6°28'28.35"E | 2014 | male | 75 | A |
| 14sm19 | Rees (lower Rhine) | 51°41'8.44"N 6°28'28.35"E | 2014 | male | 96 | A |
| 16lf02 | Rees (lower Rhine) | 51°41'8.44"N 6°28'28.35"E | 2016 | female | 83 | A |
| 16lf03 | Rees (lower Rhine) | 51°41'8.44"N 6°28'28.35"E | 2016 | female | 72 | A |
| 16lf04 | Rees (lower Rhine) | 51°41'8.44"N 6°28'28.35"E | 2016 | female | 67 | A |
| 16lf05 | Rees (lower Rhine) | 51°41'8.44"N 6°28'28.35"E | 2016 | female | 64 | A |
| 16lf06 | Rees (lower Rhine) | 51°41'8.44"N 6°28'28.35"E | 2016 | female | 78 | A |
| 16lf07 | Rees (lower Rhine) | 51°41'8.44"N 6°28'28.35"E | 2016 | female | 91 | A |
| 16lf08 | Rees (lower Rhine) | 51°41'8.44"N 6°28'28.35"E | 2016 | female | 80 | A |
| 16lf09 | Rees (lower Rhine) | 51°41'8.44"N 6°28'28.35"E | 2016 | female | 102 | C |
| 16lf10 | Rees (lower Rhine) | 51°41'8.44"N 6°28'28.35"E | 2016 | female | 75 | A |
| 16lm01 | Rees (lower Rhine) | 51°41'8.44"N 6°28'28.35"E | 2016 | male | 110 | A |
| 16lm02 | Rees (lower Rhine) | 51°41'8.44"N 6°28'28.35"E | 2016 | male | 57 | A |
| 16lm03 | Rees (lower Rhine) | 51°41'8.44"N 6°28'28.35"E | 2016 | male | 64 | A |
| 16lm04 | Rees (lower Rhine) | 51°41'8.44"N 6°28'28.35"E | 2016 | male | 84 | A |
| 16lm05 | Rees (lower Rhine) | 51°41'8.44"N 6°28'28.35"E | 2016 | male | 74 | A |
| 16lm06 | Rees (lower Rhine) | 51°41'8.44"N 6°28'28.35"E | 2016 | male | 88 | C |
| 16lm07 | Rees (lower Rhine) | 51°41'8.44"N 6°28'28.35"E | 2016 | male | 101 | C |
| 16lm08 | Rees (lower Rhine) | 51°41'8.44"N 6°28'28.35"E | 2016 | male | 70 | A |
| 16lm09 | Rees (lower Rhine) | 51°41'8.44"N 6°28'28.35"E | 2016 | male | 122 | A |
| 16lm10 | Rees (lower Rhine) | 51°41'8.44"N 6°28'28.35"E | 2016 | male | 98 | A |
| 16sf01 | Rees (lower Rhine) | 51°41'8.44"N 6°28'28.35"E | 2016 | female | 64 | A |
| 16sf02 | Rees (lower Rhine) | 51°41'8.44"N 6°28'28.35"E | 2016 | female | 81 | A |
| 16sf06 | Rees (lower Rhine) | 51°41'8.44"N 6°28'28.35"E | 2016 | female | 64 | A |
| 16sf07 | Rees (lower Rhine) | 51°41'8.44"N 6°28'28.35"E | 2016 | female | 67 | A |
| 16sf09 | Rees (lower Rhine) | 51°41'8.44"N 6°28'28.35"E | 2016 | female | 100 | A |
| 16sf12 | Rees (lower Rhine) | 51°41'8.44"N 6°28'28.35"E | 2016 | female | 56 | A |
| 16sf15 | Rees (lower Rhine) | 51°41'8.44"N 6°28'28.35"E | 2016 | female | 91 | A |
| 16sf22 | Rees (lower Rhine) | 51°41'8.44"N 6°28'28.35"E | 2016 | female | 71 | A |
| 16sm01 | Rees (lower Rhine) | 51°41'8.44"N 6°28'28.35"E | 2016 | male | 57 | A |
| 16sm02 | Rees (lower Rhine) | 51°41'8.44"N 6°28'28.35"E | 2016 | male | 74 | A |
| 16sm03 | Rees (lower Rhine) | 51°41'8.44"N 6°28'28.35"E | 2016 | male | 56 | A |
| 16sm05 | Rees (lower Rhine) | 51°41'8.44"N 6°28'28.35"E | 2016 | male | 62 | B |
| 16sm06 | Rees (lower Rhine) | 51°41'8.44"N 6°28'28.35"E | 2016 | male | 67 | B |
| 16sm07 | Rees (lower Rhine) | 51°41'8.44"N 6°28'28.35"E | 2016 | male | 117 | C |
| 16sm08 | Rees (lower Rhine) | 51°41'8.44"N 6°28'28.35"E | 2016 | male | 87 | A |
| 16sm09 | Rees (lower Rhine) | 51°41'8.44"N 6°28'28.35"E | 2016 | male | 112 | A |
| 16sm14 | Rees (lower Rhine) | 51°41'8.44"N 6°28'28.35"E | 2016 | male | 79 | A |
| 16sm18 | Rees (lower Rhine) | 51°41'8.44"N 6°28'28.35"E | 2016 | male | 83 | A |
| Nm2018 | Rees (lower Rhine) | 51°41'8.44"N 6°28'28.35"E | 2018 | na | na | C |
| 18fm01 | Rees (lower Rhine) | 51°41'8.44"N 6°28'28.35"E | 2018 | male | 106 | A |
| 18fm02 | Rees (lower Rhine) | 51°41'8.44"N 6°28'28.35"E | 2018 | male | 131 | A |
| 18fm03 | Rees (lower Rhine) | 51°41'8.44"N 6°28'28.35"E | 2018 | male | 84,41 | A |
| 18fm04 | Rees (lower Rhine) | 51°41'8.44"N 6°28'28.35"E | 2018 | male | 84,2 | A |
| 18fm05 | Rees (lower Rhine) | 51°41'8.44"N 6°28'28.35"E | 2018 | male | 128,71 | A |
| 18fm07 | Rees (lower Rhine) | 51°41'8.44"N 6°28'28.35"E | 2018 | male | 129 | A |
| 18fm08 | Rees (lower Rhine) | 51°41'8.44"N 6°28'28.35"E | 2018 | male | 109,07 | A |
| 18fm09 | Rees (lower Rhine) | 51°41'8.44"N 6°28'28.35"E | 2018 | male | 99 | A |
| 18fm10 | Rees (lower Rhine) | 51°41'8.44"N 6°28'28.35"E | 2018 | male | 83 | A |
| 18fm18 | Rees (lower Rhine) | 51°41'8.44"N 6°28'28.35"E | 2018 | female | 108 | A |
| 18nm01 | Rees (lower Rhine) | 51°41'8.44"N 6°28'28.35"E | 2018 | male | 88 | A |
| 18nm02 | Rees (lower Rhine) | 51°41'8.44"N 6°28'28.35"E | 2018 | male | 85 | A |
| 18nm03 | Rees (lower Rhine) | 51°41'8.44"N 6°28'28.35"E | 2018 | male | 63 | A |
| 18nm04 | Rees (lower Rhine) | 51°41'8.44"N 6°28'28.35"E | 2018 | male | 123 | A |
| 18nm05 | Rees (lower Rhine) | 51°41'8.44"N 6°28'28.35"E | 2018 | male | 89 | A |
| 18nm06 | Rees (lower Rhine) | 51°41'8.44"N 6°28'28.35"E | 2018 | male | 98 | A |
| 18nm07 | Rees (lower Rhine) | 51°41'8.44"N 6°28'28.35"E | 2018 | male | 67,32 | A |
| 18nm11 | Rees (lower Rhine) | 51°41'8.44"N 6°28'28.35"E | 2018 | male | 92 | A |
| 18nm12 | Rees (lower Rhine) | 51°41'8.44"N 6°28'28.35"E | 2018 | male | 85 | A |
| 18nm13 | Rees (lower Rhine) | 51°41'8.44"N 6°28'28.35"E | 2018 | male | 75,16 | A |
| 20fm01 | Rees (lower Rhine) | 51°41'8.44"N 6°28'28.35"E | 2020 | male | 90,31 | A |
| 20fm03 | Rees (lower Rhine) | 51°41'8.44"N 6°28'28.35"E | 2020 | male | 101,66 | A |
| 20fm04 | Rees (lower Rhine) | 51°41'8.44"N 6°28'28.35"E | 2020 | male | 122 | A |
| 20fm05 | Rees (lower Rhine) | 51°41'8.44"N 6°28'28.35"E | 2020 | male | 111,65 | A |
| 20fm06 | Rees (lower Rhine) | 51°41'8.44"N 6°28'28.35"E | 2020 | male | 66 | A |
| 20fm07 | Rees (lower Rhine) | 51°41'8.44"N 6°28'28.35"E | 2020 | male | 70 | A |
| 20fm08 | Rees (lower Rhine) | 51°41'8.44"N 6°28'28.35"E | 2020 | male | 89,07 | A |
| 20fm09 | Rees (lower Rhine) | 51°41'8.44"N 6°28'28.35"E | 2020 | male | 81,24 | A |
| 20fm10 | Rees (lower Rhine) | 51°41'8.44"N 6°28'28.35"E | 2020 | male | 83,88 | A |
| 20nm01 | Rees (lower Rhine) | 51°41'8.44"N 6°28'28.35"E | 2020 | male | 110 | C |
| 20nm02 | Rees (lower Rhine) | 51°41'8.44"N 6°28'28.35"E | 2020 | male | 99,77 | A |
| 20nm03 | Rees (lower Rhine) | 51°41'8.44"N 6°28'28.35"E | 2020 | male | 86,51 | C |
| 20nm04 | Rees (lower Rhine) | 51°41'8.44"N 6°28'28.35"E | 2020 | male | 79 | A |
| 20nm06 | Rees (lower Rhine) | 51°41'8.44"N 6°28'28.35"E | 2020 | male | 68 | C |
| 20nm07 | Rees (lower Rhine) | 51°41'8.44"N 6°28'28.35"E | 2020 | male | 86 | A |
| 20nm10 | Rees (lower Rhine) | 51°41'8.44"N 6°28'28.35"E | 2020 | male | 73,92 | C |
| 20nm11 | Rees (lower Rhine) | 51°41'8.44"N 6°28'28.35"E | 2020 | male | 97,45 | A |
| 20nm12 | Rees (lower Rhine) | 51°41'8.44"N 6°28'28.35"E | 2020 | male | 93,1 | A |
| 20nm13 | Rees (lower Rhine) | 51°41'8.44"N 6°28'28.35"E | 2020 | male | 74,87 | A |
| 1443 | Basel (Upper Rhine) harbour KH | 47°34'56.38"N 7°35'26.73"E | 2012 | na | na | A |
| 1444 | Basel (Upper Rhine) harbour KH | 47°34'56.38"N 7°35'26.73"E | 2012 | na | na | A |
| 1445 | Basel (Upper Rhine) harbour KH | 47°34'56.38"N 7°35'26.73"E | 2012 | na | na | A |
| 1446 | Basel (Upper Rhine) harbour KH | 47°34'56.38"N 7°35'26.73"E | 2012 | na | na | C |
| 1447 | Basel (Upper Rhine) harbour KH | 47°34'56.38"N 7°35'26.73"E | 2012 | na | na | A |
| 1448 | Basel (Upper Rhine) harbour KH | 47°34'56.38"N 7°35'26.73"E | 2012 | na | na | A |
| 1449 | Basel (Upper Rhine) harbour KH | 47°34'56.38"N 7°35'26.73"E | 2012 | na | na | A |
| 1450 | Basel (Upper Rhine) harbour KH | 47°34'56.38"N 7°35'26.73"E | 2012 | na | na | A |
| 3051 | Basel (Upper Rhine) harbour KH | 47°34'56.38"N 7°35'26.73"E | 2015 | na | na | A |
| 3052 | Basel (Upper Rhine) harbour KH | 47°34'56.38"N 7°35'26.73"E | 2015 | na | na | A |
| 3053 | Basel (Upper Rhine) harbour KH | 47°34'56.38"N 7°35'26.73"E | 2015 | na | na | A |
| 3054 | Basel (Upper Rhine) harbour KH | 47°34'56.38"N 7°35'26.73"E | 2015 | na | na | A |
| 3055 | Basel (Upper Rhine) harbour KH | 47°34'56.38"N 7°35'26.73"E | 2015 | na | na | A |
| 3127 | Basel (Upper Rhine) harbour KH | 47°34'56.38"N 7°35'26.73"E | 2015 | na | na | A |
| 3128 | Basel (Upper Rhine) harbour KH | 47°34'56.38"N 7°35'26.73"E | 2015 | na | na | A |
| 3129 | Basel (Upper Rhine) harbour KH | 47°34'56.38"N 7°35'26.73"E | 2015 | na | na | A |
| 3130 | Basel (Upper Rhine) harbour KH | 47°34'56.38"N 7°35'26.73"E | 2015 | na | na | A |
| 3131 | Basel (Upper Rhine) harbour KH | 47°34'56.38"N 7°35'26.73"E | 2015 | na | na | A |
| 4245 | Basel (Upper Rhine) harbour KH | 47°34'56.38"N 7°35'26.73"E | 2017 | na | na | A |
| 4246 | Basel (Upper Rhine) harbour KH | 47°34'56.38"N 7°35'26.73"E | 2017 | na | na | A |
| 4247 | Basel (Upper Rhine) harbour KH | 47°34'56.38"N 7°35'26.73"E | 2017 | na | na | A |
| 4248 | Basel (Upper Rhine) harbour KH | 47°34'56.38"N 7°35'26.73"E | 2017 | na | na | A |
| 4249 | Basel (Upper Rhine) harbour KH | 47°34'56.38"N 7°35'26.73"E | 2017 | na | na | A |
| 4345 | Basel (Upper Rhine) harbour KH | 47°34'56.38"N 7°35'26.73"E | 2017 | na | na | A |
| 4346 | Basel (Upper Rhine) harbour KH | 47°34'56.38"N 7°35'26.73"E | 2017 | na | na | A |
| 4347 | Basel (Upper Rhine) harbour KH | 47°34'56.38"N 7°35'26.73"E | 2017 | na | na | A |
| 4348 | Basel (Upper Rhine) harbour KH | 47°34'56.38"N 7°35'26.73"E | 2017 | na | na | A |
| 4349 | Basel (Upper Rhine) harbour KH | 47°34'56.38"N 7°35'26.73"E | 2017 | na | na | A |
| 4805 | Basel (Upper Rhine) harbour KH | 47°34'56.38"N 7°35'26.73"E | 2020 | na | na | A |
| 4806 | Basel (Upper Rhine) harbour KH | 47°34'56.38"N 7°35'26.73"E | 2020 | na | na | B |
| 4807 | Basel (Upper Rhine) harbour KH | 47°34'56.38"N 7°35'26.73"E | 2020 | na | na | A |
| 4808 | Basel (Upper Rhine) harbour KH | 47°34'56.38"N 7°35'26.73"E | 2020 | na | na | A |
| 4809 | Basel (Upper Rhine) harbour KH | 47°34'56.38"N 7°35'26.73"E | 2020 | na | na | A |
| 4810 | Basel (Upper Rhine) harbour KH | 47°34'56.38"N 7°35'26.73"E | 2020 | na | na | A |
| 4811 | Basel (Upper Rhine) harbour KH | 47°34'56.38"N 7°35'26.73"E | 2020 | na | na | A |
| 4812 | Basel (Upper Rhine) harbour KH | 47°34'56.38"N 7°35'26.73"E | 2020 | na | na | A |
| 4813 | Basel (Upper Rhine) harbour KH | 47°34'56.38"N 7°35'26.73"E | 2020 | na | na | A |
| 4814 | Basel (Upper Rhine) harbour KH | 47°34'56.38"N 7°35'26.73"E | 2020 | na | na | B |
| 4956 | Basel (Upper Rhine) St. Alban Fähre | 47°33'21.59"N 7°36' 8.96"E | 2015 | na | na | A |
| 4957 | Basel (Upper Rhine) St. Alban Fähre | 47°33'21.59"N 7°36' 8.96"E | 2015 | na | na | A |
| 4958 | Basel (Upper Rhine) St. Alban Fähre | 47°33'21.59"N 7°36' 8.96"E | 2015 | na | na | A |
| 4959 | Basel (Upper Rhine) St. Alban Fähre | 47°33'21.59"N 7°36' 8.96"E | 2015 | na | na | A |
| 4960 | Basel (Upper Rhine) St. Alban Fähre | 47°33'21.59"N 7°36' 8.96"E | 2015 | na | na | A |
| 4961 | Basel (Upper Rhine) St. Alban Fähre | 47°33'21.59"N 7°36' 8.96"E | 2015 | na | na | A |
| 5003 | Basel (Upper Rhine) St. Alban Fähre | 47°33'21.59"N 7°36' 8.96"E | 2017 | na | na | C |
| 5004 | Basel (Upper Rhine) St. Alban Fähre | 47°33'21.59"N 7°36' 8.96"E | 2017 | na | na | A |
| 5005 | Basel (Upper Rhine) St. Alban Fähre | 47°33'21.59"N 7°36' 8.96"E | 2017 | na | na | A |
| 5006 | Basel (Upper Rhine) St. Alban Fähre | 47°33'21.59"N 7°36' 8.96"E | 2017 | na | na | C |
| 5007 | Basel (Upper Rhine) St. Alban Fähre | 47°33'21.59"N 7°36' 8.96"E | 2017 | na | na | A |
| 5008 | Basel (Upper Rhine) St. Alban Fähre | 47°33'21.59"N 7°36' 8.96"E | 2017 | na | na | A |
| 3994 | Basel (Upper Rhine) Kaiseraugst | 47°32'27.82"N 7°43'13.83"E | 2017 | na | na | A |
| 3995 | Basel (Upper Rhine) Kaiseraugst | 47°32'27.82"N 7°43'13.83"E | 2017 | na | na | A |
| 3996 | Basel (Upper Rhine) Kaiseraugst | 47°32'27.82"N 7°43'13.83"E | 2017 | na | na | A |
| 3991 | Basel (Upper Rhine) Kaiseraugst | 47°32'27.82"N 7°43'13.83"E | 2017 | na | na | A |
| 4041 | Basel (Upper Rhine) Kaiseraugst | 47°32'27.82"N 7°43'13.83"E | 2017 | na | na | A |
| 4042 | Basel (Upper Rhine) Kaiseraugst | 47°32'27.82"N 7°43'13.83"E | 2017 | na | na | A |
| 4043 | Basel (Upper Rhine) Kaiseraugst | 47°32'27.82"N 7°43'13.83"E | 2017 | na | na | A |
| 4044 | Basel (Upper Rhine) Kaiseraugst | 47°32'27.82"N 7°43'13.83"E | 2017 | na | na | A |
| 4045 | Basel (Upper Rhine) Kaiseraugst | 47°32'27.82"N 7°43'13.83"E | 2017 | na | na | A |
| 4046 | Basel (Upper Rhine) Kaiseraugst | 47°32'27.82"N 7°43'13.83"E | 2017 | na | na | A |
| 90203 | Deggendorf (Donau) | 48° 47' 56.84" N 12° 59' 14.13" E | 2010 | na | na | A |
| 90204 | Deggendorf (Donau) | 48° 47' 56.84" N 12° 59' 14.13" E | 2010 | na | na | A |
| 90205 | Deggendorf (Donau) | 48° 47' 56.84" N 12° 59' 14.13" E | 2010 | na | na | A |
| 90206 | Deggendorf (Donau) | 48° 47' 56.84" N 12° 59' 14.13" E | 2010 | na | na | A |
| 90207 | Deggendorf (Donau) | 48° 47' 56.84" N 12° 59' 14.13" E | 2010 | na | na | A |
| 90208 | Deggendorf (Donau) | 48° 47' 56.84" N 12° 59' 14.13" E | 2010 | na | na | A |
| 90209 | Deggendorf (Donau) | 48° 47' 56.84" N 12° 59' 14.13" E | 2010 | na | na | A |
| 90210 | Deggendorf (Donau) | 48° 47' 56.84" N 12° 59' 14.13" E | 2010 | na | na | A |
| 90211 | Deggendorf (Donau) | 48° 47' 56.84" N 12° 59' 14.13" E | 2010 | na | na | A |
| 90212 | Deggendorf (Donau) | 48° 47' 56.84" N 12° 59' 14.13" E | 2010 | na | na | A |
| 90213 | Deggendorf (Donau) | 48° 47' 56.84" N 12° 59' 14.13" E | 2010 | na | na | A |
| 90214 | Deggendorf (Donau) | 48° 47' 56.84" N 12° 59' 14.13" E | 2010 | na | na | A |
| 90215 | Deggendorf (Donau) | 48° 47' 56.84" N 12° 59' 14.13" E | 2010 | na | na | A |
| 90216 | Deggendorf (Donau) | 48° 47' 56.84" N 12° 59' 14.13" E | 2010 | na | na | A |
| 90217 | Deggendorf (Donau) | 48° 47' 56.84" N 12° 59' 14.13" E | 2010 | na | na | A |
| 90218 | Deggendorf (Donau) | 48° 47' 56.84" N 12° 59' 14.13" E | 2010 | na | na | A |
| 90219 | Deggendorf (Donau) | 48° 47' 56.84" N 12° 59' 14.13" E | 2010 | na | na | A |
| 90220 | Deggendorf (Donau) | 48° 47' 56.84" N 12° 59' 14.13" E | 2010 | na | na | A |
| 90221 | Deggendorf (Donau) | 48° 47' 56.84" N 12° 59' 14.13" E | 2010 | na | na | A |
| 90222 | Deggendorf (Donau) | 48° 47' 56.84" N 12° 59' 14.13" E | 2010 | na | na | A |
| 90223 | Deggendorf (Donau) | 48° 47' 56.84" N 12° 59' 14.13" E | 2010 | na | na | A |
| 90224 | Deggendorf (Donau) | 48° 47' 56.84" N 12° 59' 14.13" E | 2010 | na | na | A |
| 90225 | Deggendorf (Donau) | 48° 47' 56.84" N 12° 59' 14.13" E | 2010 | na | na | A |
| 90226 | Deggendorf (Donau) | 48° 47' 56.84" N 12° 59' 14.13" E | 2010 | na | na | A |
| 90227 | Deggendorf (Donau) | 48° 47' 56.84" N 12° 59' 14.13" E | 2010 | na | na | A |
| 90228 | Deggendorf (Donau) | 48° 47' 56.84" N 12° 59' 14.13" E | 2010 | na | na | A |
| 90229 | Deggendorf (Donau) | 48° 47' 56.84" N 12° 59' 14.13" E | 2010 | na | na | A |
| 90230 | Deggendorf (Donau) | 48° 47' 56.84" N 12° 59' 14.13" E | 2010 | na | na | A |
| 90231 | Deggendorf (Donau) | 48° 47' 56.84" N 12° 59' 14.13" E | 2010 | na | na | A |
| 90232 | Deggendorf (Donau) | 48° 47' 56.84" N 12° 59' 14.13" E | 2010 | na | na | A |
| 90706 | Straubing (Donau) | 48° 54' 4.98" N 12° 37' 15.57" E | 2010 | na | na | A |
| 90707 | Straubing (Donau) | 48° 54' 4.98" N 12° 37' 15.57" E | 2010 | na | na | A |
| 90708 | Straubing (Donau) | 48° 54' 4.98" N 12° 37' 15.57" E | 2010 | na | na | A |
| 90709 | Straubing (Donau) | 48° 54' 4.98" N 12° 37' 15.57" E | 2010 | na | na | A |
| 90710 | Straubing (Donau) | 48° 54' 4.98" N 12° 37' 15.57" E | 2010 | na | na | A |
| 90711 | Straubing (Donau) | 48° 54' 4.98" N 12° 37' 15.57" E | 2010 | na | na | A |
| 90712 | Straubing (Donau) | 48° 54' 4.98" N 12° 37' 15.57" E | 2010 | na | na | A |
| 90713 | Straubing (Donau) | 48° 54' 4.98" N 12° 37' 15.57" E | 2010 | na | na | A |
| 90714 | Straubing (Donau) | 48° 54' 4.98" N 12° 37' 15.57" E | 2010 | na | na | A |
| 90715 | Straubing (Donau) | 48° 54' 4.98" N 12° 37' 15.57" E | 2010 | na | na | A |
| 90716 | Straubing (Donau) | 48° 54' 4.98" N 12° 37' 15.57" E | 2010 | na | na | A |
| 90717 | Straubing (Donau) | 48° 54' 4.98" N 12° 37' 15.57" E | 2010 | na | na | A |
| 90718 | Straubing (Donau) | 48° 54' 4.98" N 12° 37' 15.57" E | 2010 | na | na | A |
| 90719 | Straubing (Donau) | 48° 54' 4.98" N 12° 37' 15.57" E | 2010 | na | na | A |
| 90720 | Straubing (Donau) | 48° 54' 4.98" N 12° 37' 15.57" E | 2010 | na | na | A |
| 90721 | Straubing (Donau) | 48° 54' 4.98" N 12° 37' 15.57" E | 2010 | na | na | A |
| 90722 | Straubing (Donau) | 48° 54' 4.98" N 12° 37' 15.57" E | 2010 | na | na | A |
| 90723 | Straubing (Donau) | 48° 54' 4.98" N 12° 37' 15.57" E | 2010 | na | na | A |
| 90724 | Straubing (Donau) | 48° 54' 4.98" N 12° 37' 15.57" E | 2010 | na | na | A |
| 90725 | Straubing (Donau) | 48° 54' 4.98" N 12° 37' 15.57" E | 2010 | na | na | A |
| 90726 | Straubing (Donau) | 48° 54' 4.98" N 12° 37' 15.57" E | 2010 | na | na | A |
| 90727 | Straubing (Donau) | 48° 54' 4.98" N 12° 37' 15.57" E | 2010 | na | na | A |
| 90728 | Straubing (Donau) | 48° 54' 4.98" N 12° 37' 15.57" E | 2010 | na | na | A |
| 90729 | Straubing (Donau) | 48° 54' 4.98" N 12° 37' 15.57" E | 2010 | na | na | A |
| 90730 | Straubing (Donau) | 48° 54' 4.98" N 12° 37' 15.57" E | 2010 | na | na | A |
| 90731 | Straubing (Donau) | 48° 54' 4.98" N 12° 37' 15.57" E | 2010 | na | na | A |
| 90732 | Straubing (Donau) | 48° 54' 4.98" N 12° 37' 15.57" E | 2010 | na | na | A |
| 90733 | Straubing (Donau) | 48° 54' 4.98" N 12° 37' 15.57" E | 2010 | na | na | A |
| 90734 | Straubing (Donau) | 48° 54' 4.98" N 12° 37' 15.57" E | 2010 | na | na | A |
| 90735 | Straubing (Donau) | 48° 54' 4.98" N 12° 37' 15.57" E | 2010 | na | na | A |
| NM00TT | Trave | 53°54'21.87"N 10°43'14.78"E | 2016 | na | na | C |
| NM49LT | Trave | 53°54'21.87"N 10°43'14.78"E | 2016 | na | na | C |
| NM51LT | Trave | 53°54'21.87"N 10°43'14.78"E | 2016 | na | na | B |
| NM53LT | Trave | 53°54'21.87"N 10°43'14.78"E | 2016 | na | na | C |
| NM55LT | Trave | 53°54'21.87"N 10°43'14.78"E | 2016 | na | na | B |
| NM57LT | Trave | 53°54'21.87"N 10°43'14.78"E | 2016 | na | na | C |
| NM58LT | Trave | 53°54'21.87"N 10°43'14.78"E | 2016 | na | na | C |
| NM62LT | Trave | 53°54'21.87"N 10°43'14.78"E | 2016 | na | na | C |
| NM67LT | Trave | 53°54'21.87"N 10°43'14.78"E | 2016 | na | na | C |
| NM69LT | Trave | 53°54'21.87"N 10°43'14.78"E | 2016 | na | na | B |
| NM73HT | Trave | 53°54'21.87"N 10°43'14.78"E | 2016 | na | na | C |
| NM74HT | Trave | 53°54'21.87"N 10°43'14.78"E | 2016 | na | na | C |
| NM80HT | Trave | 53°54'21.87"N 10°43'14.78"E | 2016 | na | na | B |
| NM82HT | Trave | 53°54'21.87"N 10°43'14.78"E | 2016 | na | na | C |
| Nm84HT | Trave | 53°54'21.87"N 10°43'14.78"E | 2016 | na | na | C |
| NM85HT | Trave | 53°54'21.87"N 10°43'14.78"E | 2016 | na | na | C |
| NM91HT | Trave | 53°54'21.87"N 10°43'14.78"E | 2016 | na | na | C |
| NM93HT | Trave | 53°54'21.87"N 10°43'14.78"E | 2016 | na | na | C |
| NM94HT | Trave | 53°54'21.87"N 10°43'14.78"E | 2016 | na | na | B |
| NM97LT | Trave | 53°54'21.87"N 10°43'14.78"E | 2016 | na | na | C |
