# Supplementary material for: Round gobies (Neogobius melanostomus) in the River Rhine: Population genetic support for invasion via two different routes
Source: PLoS One. 2024 Sep 19;19(9):e0310692. doi: 10.1371/journal.pone.0310692 (PMC11412513; doi:10.1371/journal.pone.0310692)
Supplement: S2 Table — (DOCX) [file pone.0310692.s002.docx]

**Table S1:** *Neogobius melanostomus* - dloop haplotypes alignment

....|....| ....|....| ....|....| ....|....| ....|....|

5 15 25 35 45

Allele A AGAAGGGAGA TTTTAACTCC CACCCCTAAC TCCCAAAGCT AGAATTCTAA

Allele B AGAAGGGAGA TTTTAACTCC CACCCCTAAC TCCCAAAGCT AGAATTCTAA

Allele C AGAAGGGAGA TTTTAACTCC CGCCCCTAAC TCCCAAAGCT AGAATTCTAA

....|....| ....|....| ....|....| ....|....| ....|....|

55 65 75 85 95

Allele A ATTTAACTAT TCTTTGTACA TATAATAATG TTCTAAAATA TATAT-----

Allele B ATTTAACTAT TCTTTGTACA TATAATAATG TTCTAAAATA TATAT-----

Allele C ATTTAACTAT TCTTTGTACA TATAATAATG TTCTATAATA TATATATATA

....|....| ....|....| ....|....| ....|....| ....|....|

105 115 125 135 145

Allele A -GTAT-ATCA CCATTAATAG ACTTTAACCA TTCAAGAGTA CATATATATA

Allele B -GTATTATCA CCATTAATAG ACTTTAACCA TTCAAGAGTA CATATATATA

Allele C TGTACTATCA CCATTAATAG ACTTTAACCA TTCAAGAGTA CATATATATA

....|....| ....|....| ....|....| ....|....| ....|....|

155 165 175 185 195

Allele A TGTTTTATCA ACATATATAT ATATATTAAC CATATATAAT AATGTTCTAG

Allele B TGTTTTATCA ACATATATAT ATAT--TAAC CATATATAAT AATGTTCTAG

Allele C TGTTTTATCA ACATATATAT ATAT--TAAC CATATATAAT AATGTTCTAG

....|....| ....|....| ....|....| ....|....| ....|....|

205 215 225 235 245

Allele A GACATATATG TATTATCACC ATTAATAGAC TTTAACCATT CAAGAGTACA

Allele B GACATATATG TATTATCACC ATTAATAGAC TTTAACCATT CAAGAGTACA

Allele C GACATATATG TATTATCACC ATTAATAGAC TTTAACCATT CAAGAGTACA

....|....| ....|....| ....|....| ....|....| ....|....|

255 265 275 285 295

Allele A TATATATATG TTTTATCAAC ATATATATAT ATTAACCATA TATAATAATG

Allele B TATATATATG TTTTATCAAC ATATATATAT ATTAACCATA TATAATAATG

Allele C TATATATATG TTTTATCAAC ATATATATAT ATTAACCATA TATAATAATG

....|....| ....|....| ....|....| ....|....| ....|....|

305 315 325 335 345

Allele A TTCTAGGACA TATATGTATT ATCACCATTA ATAGACTTTG ACCATTCAAG

Allele B TTCTAGGACA TATATGTATT ATCACCATTA ATAGACTTTA ACCATTCAAG

Allele C TTCTAGGACA TATATGTATT ATCACCATTA ATAGACTTTA ACCATTCAAG

....|....| ....|....| ....|....| ....|....| ....|....|

355 365 375 385 395

Allele A AGTACATATA TATATGTTTT ATCAACATAT ATATATATTA ACCATATATA

Allele B AGTACATATA TATATGTTTT ATCAACATAT ATATATATTA ACCATATATA

Allele C AGTACATATA TATATGTTTT ATCAACATAT ATATATATTA ACCATATATA

....|....| ....|....| ....|....| ....|....| ....|....|

405 415 425 435 445

Allele A ATAATGCTTT AAGATATATA TGTATTATCA CCATTAATAG ACTTTAACCA

Allele B ATAATGTTCT AGGACATATA TGTATTATCA CCATTAATAG ACTTTAACCA

Allele C ATAATGTTCT AGGACATATA TGTATTATCA CCATTAATAG ACTTTAACCA

....|....| ....|....| ....|....| ....|....| ....|....|

455 465 475 485 495

Allele A TTCAAGAGTA CATATATATG AATGTACCAT CAACATATAT ATATATAAAC

Allele B TTCAAGAGTA CATATGTATG AATGTACTAT CAACATATAT ATATATAAAC

Allele C TTCAAGAGTA CATATATATG AATGTACTAT CAACATATAT ATATATAAAC

....|....| ....|....| ....|....| ....|....| ....|....|

505 515 525 535 545

Allele A CATATATAAT AATGCTTTAA GATATATATG TATTATCACC ATTAATAGAT

Allele B CATATATAAT AATGCTTTAA GATATATATG TATTATCACC ATTAATAGAT

Allele C CATATATAAT AATGCTTTAA GATATATATG TATTATCATC ATTAATAGAT

....|....| ....|....| ....|....| ....|....| ....|....|

555 565 575 585 595

Allele A TTT-AGCCAT TCATTCATCA ACAATCATTC AAGAATGCAA AATACATTTA

Allele B TTT-AGCCAT TCATTCATCA ACAATCATTC AAGAATGCAA AATACATTTA

Allele C TTTTAGCCAT TCATTCATCA ACAATCATTC AAGAATGCAA AATACATTTA

....|....| ....|....| ....|....| ....|....| ....|....|

605 615 625 635 645

Allele A TTTAATATCA ACAAAAATGC CTGATAATAG AAATTACCCA ATTAAATAAC

Allele B TTTAATATCA ACAAAAATGC CTGATAATAG AAATTACCCA ATTAAATATC

Allele C TTTAATATCA ACAAAAATGC CTGATAATAG AAATTACCCA ATTAAATATC

....|..

655

Allele A CTCACAC

Allele B CTCACAC

Allele C CTCACAC
